# Dynamic Modulation of Microglia/Macrophage Polarization by miR-124 after Focal Cerebral Ischemia

**DOI:** 10.1007/s11481-016-9700-y

**Published:** 2016-08-18

**Authors:** Somayyeh Hamzei Taj, Widuri Kho, Markus Aswendt, Franziska M. Collmann, Claudia Green, Joanna Adamczak, Annette Tennstaedt, Mathias Hoehn

**Affiliations:** 1 In-vivo-NMR Laboratory, Max Planck Institute for Metabolism Research, Gleuelerstrasse 50, D-50931 Köln, Germany; 2 Department of Radiology, Leiden University Medical Center, Leiden, Netherlands; 3 Percuros BV, Enschede, Netherlands

**Keywords:** Stroke, Microglia/macrophage polarization, Pro-inflammatory and anti-inflammatory phenotypes, miRNA-124, Neuroinflammation

## Abstract

Mononuclear phagocytes respond to ischemic stroke dynamically, undergoing an early anti-inflammatory and protective phenotype followed by the pro-inflammatory and detrimental type. These dual roles of microglia/macrophages suggest the need of subtle adjustment of their polarization state instead of broad suppression. The most abundant brain-specific miRNA, miR-124, promotes neuronal differentiation but can also modulate microglia activation and keeps them in a quiescent state. We addressed whether the intracerebral injection of miR-124 in a mouse model of ischemic stroke before or after the peak phase of the pro-inflammatory polarization modifies the pro−/anti- inflammatory balance. In the sub-acute phase, 48 h after stroke, liposomated miR-124 shifted the predominantly pro-inflammatory polarized microglia/macrophages toward the anti-inflammatory phenotype. The altered immune response improved neurological deficit at day 6 after stroke. When miR-124 was injected 10 days after stroke, the pro−/anti- inflammatory ratio was still significantly reduced although to a lower degree and had no effect on recovery at day 14. This study indicates that miR-124 administration before the peak of the pro-inflammatory process of stroke is most effective in support of increasing the rehabilitation opportunity in the sub-acute phases of stroke. Our findings highlight the important role of immune cells after stroke and the therapeutic relevance of their polarization balance.

**Electronic supplementary material:**

The online version of this article (doi:10.1007/s11481-016-9700-y) contains supplementary material, which is available to authorized users.

## Introduction

Ischemic stroke is a major cause of death and disability worldwide (Donnan et al. 2008), but presently available therapies only benefit a small fraction of all patients. In stroke, primary cell death is directly caused by ischemia, but is followed by secondary deterioration due to inflammatory response to the primary event (Dirnagl et al. 1999; Jin et al. 2010). Next to neutrophils, granulocytes and T-cells infiltrating at day 2–4 after stroke, the early recruitment of central nervous system (CNS) resident microglia and infiltrating macrophages at day 1–2 determines strongly the outcome of CNS repair (Hanisch and Kettenmann 2007). However, these cells have been shown to play a protective as well as a detrimental role. They promote CNS recovery by cleaning up cell debris and releasing a variety of trophic factors and cytokines that are important for neurogenesis, axonal regeneration, angiogenesis, and vascular repair (Thored et al. 2009; Kwon et al. 2013; Hu et al. 2015). On the other hand, microglia/macrophages can aggravate tissue damage and impair functional recovery (Ekdahl et al. 2003; Miron et al. 2013). This dual role has been described as a polarization between two phenotypes, i.e. the anti-inflammatory, protective state at early stages of ischemic stroke and the pro-inflammatory, detrimental state which dominates at later stages (Hu et al. 2012). The two activation phenotypes are mainly characterized by increased protein synthesis of anti-inflammatory mediators such as interleukin-10 (IL-10), IL-4, IL-13, and tumor growth factor β (TGF-β), or pro-inflammatory mediators such as interferon γ (IFN-γ), tumor necrosis factor α (TNF-α), and IL-6 (Patel et al. 2013). In addition, pro-inflammatory factors, including iNOS, CD16, CD32, and CD86 are biomarkers of microglia/macrophages, which are regarded to be detrimental (Jia et al. 2016)(Hirai et al. 2013). In contrast, anti-inflammatory factors Arg1, CD206, CCL22, Ym1/2 are the biomarkers of microglia/macrophages, which are considered to be beneficial (Hu et al. 2012) (Liu et al. 2013). Stroke induces a systemic immunodepression, which leads to infections such as pneumonia as a leading cause of death in patients after stroke. However, preventive antibiotic therapy and broad immune suppression failed to improve clinical outcome in stroke patients (Dirnagl et al. 2007; Hu et al. 2012). Therefore, we focused on a novel class of immune modulators with clinical potential to target specific immune cell phenotypes and modify their activation state.

A key regulator of microglia quiescence and modulator of macrophage activation is miR-124 (Ponomarev et al. 2011). Similar to small interfering ribonucleic acid (siRNA), microRNAs are small non-protein coding RNAs which bind to mRNAs and lead to their direct degradation and subsequent suppression of protein translation (Bartel 2009; Bi et al. 2009). The most abundant brain-specific microRNA, miR-124, has been shown to promote neuronal differentiation in neuronal progenitor cells (Makeyev et al. 2007; Visvanathan et al. 2007). miR-124 is expressed in microglia but not in peripheral monocytes and macrophages. It affects two main transcription factors CCAAT/enhancer binding protein alpha (C/EBP-α), and its downstream target PU.1, resulting in a switch from an activated state to a non-activated CD45^low^ and major histocompatibility class (MHC) II^low^ state (Ponomarev et al. 2011). Peripheral administration of miR-124 in experimental autoimmune encephalomyelitis caused suppression of the disease by deactivation of macrophages and reduced activation of myelin-specific T-cells. Recently, we have been able to demonstrate that intracerebral administration of miR-124 after stroke has led to a clear shift of microglia/macrophages into the anti-inflammatory phenotype, as recognized by the upregulation of the marker Arg-1. This treatment resulted in a significant correlation of Arg-1 upregulation with neuronal survival and with functional improvement (Hamzei Taj et al. 2016).

These earlier results motivated us to investigate in the present study how miR-124 affects the polarization ratio of pro-inflammatory (M1) and anti-inflammatory (M2) phenotypes in microglia/macrophages after stroke. Although we are aware of the description of the states with M1 and M2 being a simplified scheme, we decided to stick to this nomenclature as it best describes our intention of shifting the status from the pro-inflammatory, detrimental towards the anti-inflammatory, protective phase. For this purpose, we selected the widely used CD16/32 as a pro-inflammatory biomarker, which is implicated in cellular cytotoxicity (Bhatnagar et al. 2014), and the mannose receptor CD206 as an anti-inflammatory biomarker, which is involved in tissue recovery and function restoration (Perego et al. 2011). We show here for the first time that intracerebral miR-124 administration after stroke results in a significant increase of microglia/macrophages with M2 phenotype, paralleled by a decrease of the M1 phenotype. This substantial decrease of the M1:M2 ratio correlates strongly with functional outcome, thus indicating the therapeutic role for miR-124 in shifting the inflammatory cells into the anti-inflammatory, protective M2 phenotype.

## Material and Methods

### Experimental Groups

The aim of this study was to investigate whether the intracerebral injection of miR-124 in a mouse model of ischemic stroke before or after the peak phase of M1 polarization modifies the M1/M2 balance. For this purpose, a total of 34 adult male C57BL6/N mice (11–12 weeks, 21–25 g; Janvier, Saint Berthevin Cedex, France) were randomly allocated to different experimental groups of miR-124 injection and survival times. Animals were housed under fixed circadian rhythm with ad libitum access to food and water. All surgical and scanning procedures were performed under Isoflurane anesthesia and core body temperature control. Stroke was induced in all mice via the middle cerebral artery occlusion (MCAO) model (cf. below). Successful ischemic stroke lesions were verified by magnetic resonance imaging (MRI) scans 48 h after MCAO, before intracranial miR-124 injection, and again before perfusion fixation at day 6 or day 14, respectively.

In Experiment 1, fifteen mice were subjected to MCAO and perfused 6 days later, divided into the following three groups: i) control mice exposed to stroke only (*n* = 5), ii) miR-treated mice which received an intracranial injection of liposomated miR-124 48 h after MCAO (*n* = 5), and iii) mice receiving a liposomated random primer injection 48 h after MCAO (n = 5) as negative control.

In Experiment 2, fourteen mice were subjected to MCAO and perfused 14 days later, divided into the following three groups: i) control mice subjected to stroke only (*n* = 5), ii) miR-treated mice which received an intracranial injection of liposomated miR-124 48 h after MCAO (*n* = 6), and iii) the negative control group that was injected with a liposomated random primer 48 h after MCAO (*n* = 3).

In Experiment 3, stroke was induced in ten mice, and they were perfused 14 days later, divided into the following two groups: control mice subjected to stroke only (*n* = 5), and the miR-treated mice with liposomated miR-124 injection at day 10 post MCAO (n = 5).

### Middle Cerebral Artery Occlusion

Transient occlusion of the right middle cerebral artery (MCAO) was induced with the intraluminal filament model, as described previously (Adamczak et al. 2014). Briefly, mice were initially anesthetized with 2 % Isoflurane (O_2_:N_2_O, 30:70 %) and were subcutaneously injected with 4 mg/kg Carprofen (Pfizer, Berlin, Germany). The body core temperature was controlled during surgery via a temperature-regulated heating pad (medres GmbH, Cologne, Germany). The right common carotid artery (CCA), external carotid artery (ECA), and internal carotid artery (ICA) were presented. A silicon rubber-coated filament with the length of 20 mm and a tip diameter of 170 μm (7017PK5Re, Doccol Corporation, Sharon, USA) was advanced into the ICA lumen until it blocked the origin of MCA. During 30 min occlusion time, mice were allowed to recover from anesthesia in a controlled heating box. Afterwards, the animals were re-anesthetized, the reperfusion was induced by filament withdrawal and the CCA was ligated. Following surgery, NaCl was subcutaneously injected in all animals twice daily until stabilization of body weight. Only the mice with cortico-striatal lesion, observed by MRI two days after MCAO, were selected for the experiments.

### Intracranial Injection of Liposomated miR-124

T2-weighted MRI was used to determine the injection coordinates 48 h after MCAO. At the day of injection the miR-124 (2 μg in 50 μl PBS, PM10691 Applied Biosystems, Carlsbad, CA, USA) or the negative control of miR (2 μg in 50 μl PBS , AM17110; Applied Biosystems) was mixed with the transfection agent Lipofectamine 2000 (Invitrogen, Paisley, UK). Intracranial injection into the right striatum, ipsilateral to the lesion, was performed as described elsewhere (Aswendt et al. 2012). Briefly, mice were fixed in a stereotactic frame (Stoelting, Dublin, Ireland), and 2.0 μl of the suspension containing 100 ng of miR or random primer was injected with a Hamilton syringe (26G needle) at the following coordinates relative to bregma: AP +0.5; L + 1.4; DV −2.4 with an infusion rate of 500 nl/min. The needle was kept in place for another 5 min before removal.

### Functional Test

To observe the different aspects of neurological functions, a set of behavioral tests was performed before and every 2 days after MCAO using the modified neurological deficit scores (mNDS), a modification of a previous report (Chen et al. 2001). The modified NDS (mNDS) has already been described recently (Hamzei Taj et al. 2016). In short, it consists of a set of motor tests (muscle status and abnormal movement), sensory tests (tactile, and proprioceptive), and reflex tests on a scale of 0–16. One point was given for the failure of a performed test or for the loss of a tested reflex. Thus, higher scores indicate higher severity of ischemia.

### Magnetic Resonance Imaging

MRI was performed using a 9.4 T Biospec animal MRI system with a 20 cm horizontal bore magnet (Bruker BioSpin, Ettlingen, Germany) equipped with actively shielded gradient coils (BGA9S, 750 mT/m, Bruker BioSpin), using ParaVision 5 software. Radio frequency (RF) transmission was achieved with a 112/72 mm od/id mouse resonator (Bruker) while signal was received with a dedicated quadrature mouse head surface coil (Bruker BioSpin). Mice were anesthetized with 2 % Isoflurane in a 30/70 oxygen/air mixture and placed in an animal holder (Bruker) using a tooth bar and ear bars for stable positioning. Respiration rate was monitored using a pressure sensitive pad placed underneath the mice. The physiological status of the animal was monitored with DASYlab software (National Instruments, Austin, TX, USA). The body core temperature was monitored with a rectal probe and was kept constant at 37 ± 1.0 °C using a water blanket connected to a feedback controlled automated temperature control unit (medres, Cologne).

Tripilot gradient-echo scans were used for definite positioning of the mouse head in the magnet. For lesion visualization, T2-weighted images were acquired with a multi-slice multi-echo (MSME) spin echo sequence (TR/TE = 5000 ms/10 ms, 16 echoes, 10 coronal slices, slice thickness 0.5 mm, FOV 2.5 × 2.5 cm^2^, matrix 256 × 256, resolution 98 × 98 μm^2^, bandwidth 75 kHz).

Quantitative T2 maps were calculated from the multi-echo trains using the IDL software (Exelis Visual Information Solutions, Boulder, CO, USA), by pixelwise fitting signal intensities to a mono-exponential decay curve. Average T2 relaxation of the healthy cortex and striatum was determined. For determination of the lesion volume, all pixels on the ipsilateral hemisphere were counted with T2 values above the threshold of normal T2 + 2 standard deviations.

### Immunohistochemistry

Animals were allowed to survive for 6 or 14 days after MCAO and were subsequently transcardially perfused under deep Isoflurane anesthesia with 20 ml cold phosphate buffered saline (PBS), followed by 20 ml 4 % paraformaldehyde (PFA). Afterwards, brains were post-fixed in 4 % PFA overnight and then cryo-protected by immersion in 30 % sucrose solution for the next 2 days at 4 °C. Then, brains were frozen in −40 °C methylbutane (Sigma-Aldrich, Taufkirchen, Germany) and subsequently stored at −80 °C. The brains were cut into 10 μm coronal slices using a cryostat (Leica Microsystems, Wetzlar, Germany), directly mounted, and stored at −20 °C.

At the day of immunostaining the cryosections were kept at room temperature (RT) for 30 min, and for stainings of ionized calcium-binding adapter molecule 1(Iba-1) acetone pre-treatment at −20 °C for 20 min was performed. To prevent non-specific binding of antibodies, the sections were pre-incubated in 5 % normal serum and 0.25 % Triton X-100, in potassium phosphate buffered saline (KPBS) for 60 min at RT. The treated sections were incubated overnight at 4 °C with subsequent primary antibodies: rabbit polyclonal anti Iba-1 (1:1000, 019–19,741; Wako Chem, Osaka, Japan), mouse anti-mannose receptor, MMR/CD206, (1:200, AF2535; R & D Systems, Minneapolis, MN, USA) and rat anti CD16/32 (1:200, 101,301, Biolegend, San Diego, CA), followed by Cy5 and Cy3 conjugated secondary antibodies (1:200, Jackson Immuno Research, West Grove, PA, USA) for 2 h at RT. For nuclear staining Hoechst 33,342 (1:1000; Invitrogen, Carlsbad, USA) was added together with secondary antibodies. Negatively stained control sections were included with equal preparation, excluding primary antibodies.

Three sections per mouse were imaged with a fluorescent microscope (BZ-9000 Keyence, Osaka, Japan) with 4×, 20× and 40× magnification objectives. 6 different regions of interest (ROIs) in each brain section were selected with the exposure time kept constant: the border and the core region of the ischemic hemisphere in the cortex and the striatum, further, two ROIs in the cortex and striatum of the intact hemisphere.

### Quantitative Immunohistochemical Analysis

To observe the polarization of microglia/macrophages, immunofluorescence images were analyzed using NIH ImageJ analysis software (ImageJ) and TissueQuest 4.0 (TissueGnostics, Vienna, Austria), a specific image cytometry analysis software. Cells were identified on the tissue section based on the nuclei staining (nuclei size, staining intensity and discrimination by area was optimized manually) followed by the analysis of specific staining. To achieve optimal cell detection the background threshold was defined and due to the coverage of Iba-1 cell ramification with total nuclei intensity, the cut-offs were defined for each ROI to discriminate false signals. Scattergrams were created to visualize the corresponding positive cells in the source ROI through the real-time back gating component. Mean intensity and the relative number of co-expressed Iba-1 and CD206 or CD16/32 were obtained, and mean values were estimated from analyses of at least three brain sections per mouse. To observe M1 and M2 representative markers in double staining with Iba-1, the above-defined 6 different ROIs provided the following number of cells/mm^3^: i) total cell density according to the nuclei staining, ii) Iba-1^+^ microglial/macrophage cell density, iii) CD206^+^/Iba-1^+^ cell density, iv) CD16/32^+^/Iba-1^+^ cell density. Then, a mean was calculated for each region for each animal.

### Statistics

Data were analyzed by SPSS version 22 (IBM SPSS statistics, Ehningen, Germany). The Normality test and homogeneity of variances were evaluated for all data. For behavioral scores (mNDS) the nonparametric analysis approach, Kruskal-Wallis H, was performed. IHC data from early time point of liposomated miR-124 injection at day 2 were tested for significant changes between the 3 groups using one-way analysis of variance (ANOVA) with Bonferroni corrected *posthoc* comparisons. Using an independent one-tailed Student’s *t*-test, we tested the data of late liposomated miR-124 injection to observe the significant changes between the stroke-only group and the group treated with miR-124 at day 10.

The IHC numbers are represented by box-and-whisker plots (Figs. 3, 4 and 6) wherein each box shows the central 50 % of the data points, the interquartile range (IQR), a horizontal line in each box indicates the median, and the vertical bars speak for the spread of 1.5 × IQR. Dots display outliers, which were included in calculations of significance. The box-and-whisker plots were generated by using SPSS version 22.

Bivariate correlation analysis between M1/M2 expression by Iba-1^+^ cells and mNDS were measured with Spearman’s correlation coefficient. Regression was done with M1/M2 expression by Iba-1^+^ cells as the independent variable and mNDS as the dependent variable. A *p*-value ≤0.05 was regarded statistically significant.

## Results

### Characterization of Ischemic Lesion after Ischemia

**Fig. 1:**
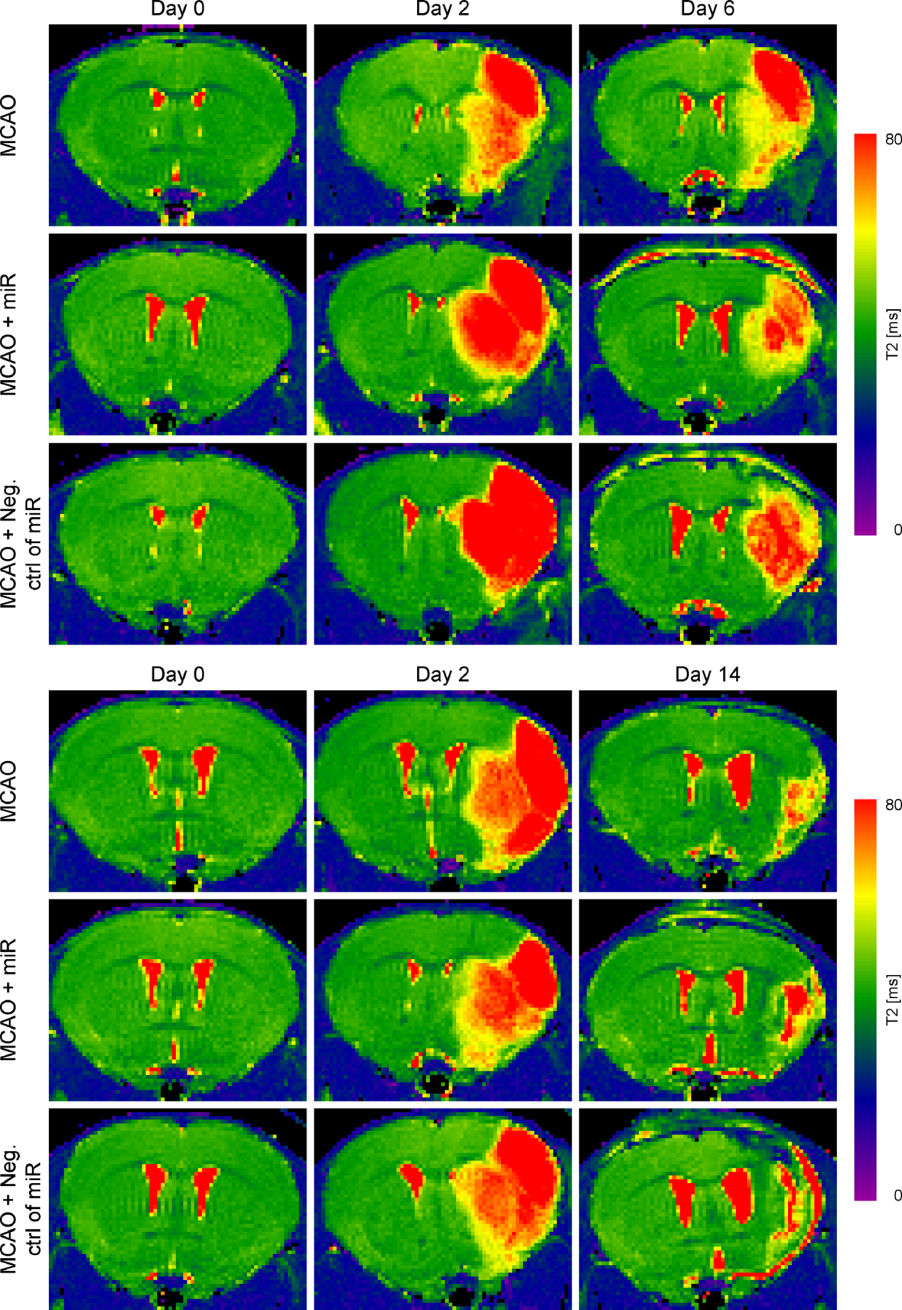
Characterization of ischemic lesion after ischemia. Representative T2map of three experimental groups subjected to MCAO and perfused at day 6 or day 14, respectively. T2 maps are shown as coronal brain section. T2 map of intact subjects, 1 week before MCAO, shows equal intensity of both hemispheres. 48 h after MCAO the cortico-striatal lesions are visible in the right hemisphere in T2-weighted images. A gradual shrinkage of infarct volume was visible at days 6 and 14 compared to day 2, showing the continuous reduction in vasogenic edema after stroke

Two characteristic lesion types were generated 1) lesions restricted to the striatum and 2) lesions involving both striatum and cortex. We excluded the striatal-only ischemic mice from further experiments and used only a homogeneous group of cortico-striatal lesions. Quantitative MRI T2 maps of the included animals were acquired before stroke induction, and at day 2 and day 6, respectively at day 2 and day 14 after MCAO, for experimental protocol 1 and 2. Thus the acute and chronic ischemic lesion location, the size and the development were detected (Fig. 1). One week before MCAO, T2 maps showed no signs of lesion and the healthy subjects presented equal T2 values of both hemisphere. At day 2 after MCAO, areas with markedly increased T2 values showed the development of the lesion extent over time. A gradual decrease in lesion volume was visible on the T2 maps at day 6 or day 14 (Fig. 1), showing that the vasogenic edema continuously resolved. A quantitative analysis of the lesion volume based on the elevated T2 relaxation time in the ischemic territory showed no significant difference between groups for all three experimental protocols (Suppl. Fig. 1).

### Morphology of Microglial/Macrophage after Stroke

First we examined the morphology of ionized calcium-binding adapter molecule 1(Iba-1) positive immune cells in the stroke core, the border and the surrounding healthy tissue. Iba-1 detects CNS resident microglia and infiltrating monocyte-derived macrophages.

**Fig. 2:**
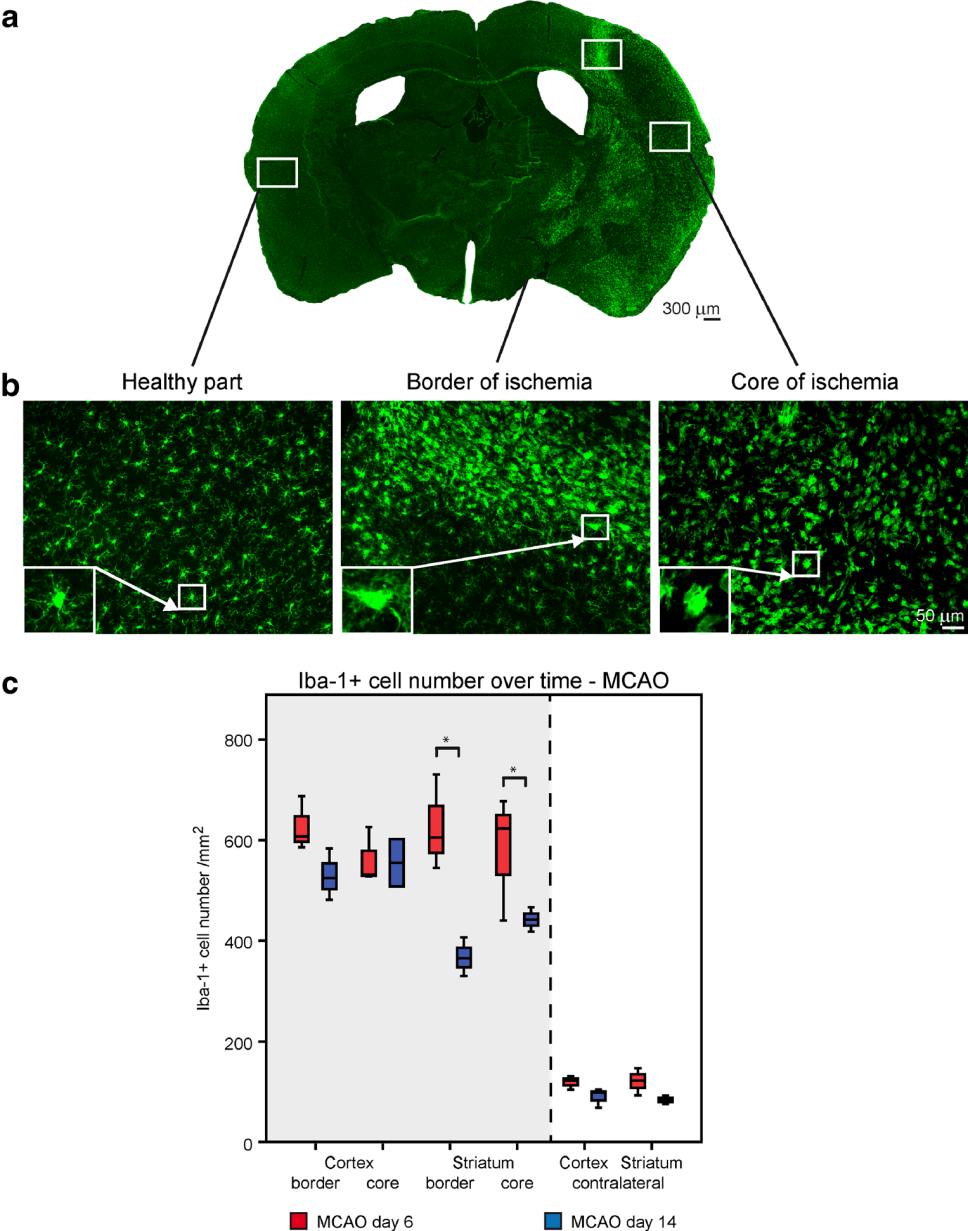
Different morphology and kinetics of microglial/macrophage after stroke. The photomicrographs show the localization of Iba-1 positive cells intensely at the ipsilateral ischemic hemisphere at day 6 after stroke in 4× magnification (**a**), and the different morphology of microglial/macrophage cells classified into ramified, intermediate, amoeboid or round phenotype in 20× magnification (**b**). Representative graph shows the number of Iba-1 positive cells in the core and border zone of cortex and striatum at day 6 in comparison to day 14 after stroke (**c**). A significant increase in Iba-1 cell number was detected at day 6 after the onset of stroke. *n* = 5 mice for each time point. Median and minimum/maximum values are shown; *P < 0.05 at independent one-tailed Student’s t-test. *Scale bar* 50 μm

Iba-1 positive cells were visible in both intact and ischemic hemisphere, although with different morphology and distribution. They were densely gathered in the ischemic hemisphere around and inside the lesion (Fig. 2a). Microglia/macrophages were categorized into ramified, intermediate, amoeboid or round phenotypes. The ramified type with long thin branching processes and a small cell body was mostly observed in the contralateral hemisphere and in the healthy parts of the ipsilateral ischemic hemisphere. In the peri-infarct area, the majority of Iba-1^+^ cells acquired the intermediate morphology with short or long swollen processes and amorphous larger cell body. In contrast, amoeboid and round shaped Iba-1^+^ cells were exclusively found in the ischemic core (Fig. 2b).

We noted a pronouncedly higher Iba-1^+^ cell density in the striatum at day 6 when compared with the results of day 14 post stroke (Fig. 2c), emphasizing the increased early stage proliferation of Iba-1^+^ cells after stroke.

### miR-124 Effects on Microglia/Macrophages after Stroke

**Fig. 3:**
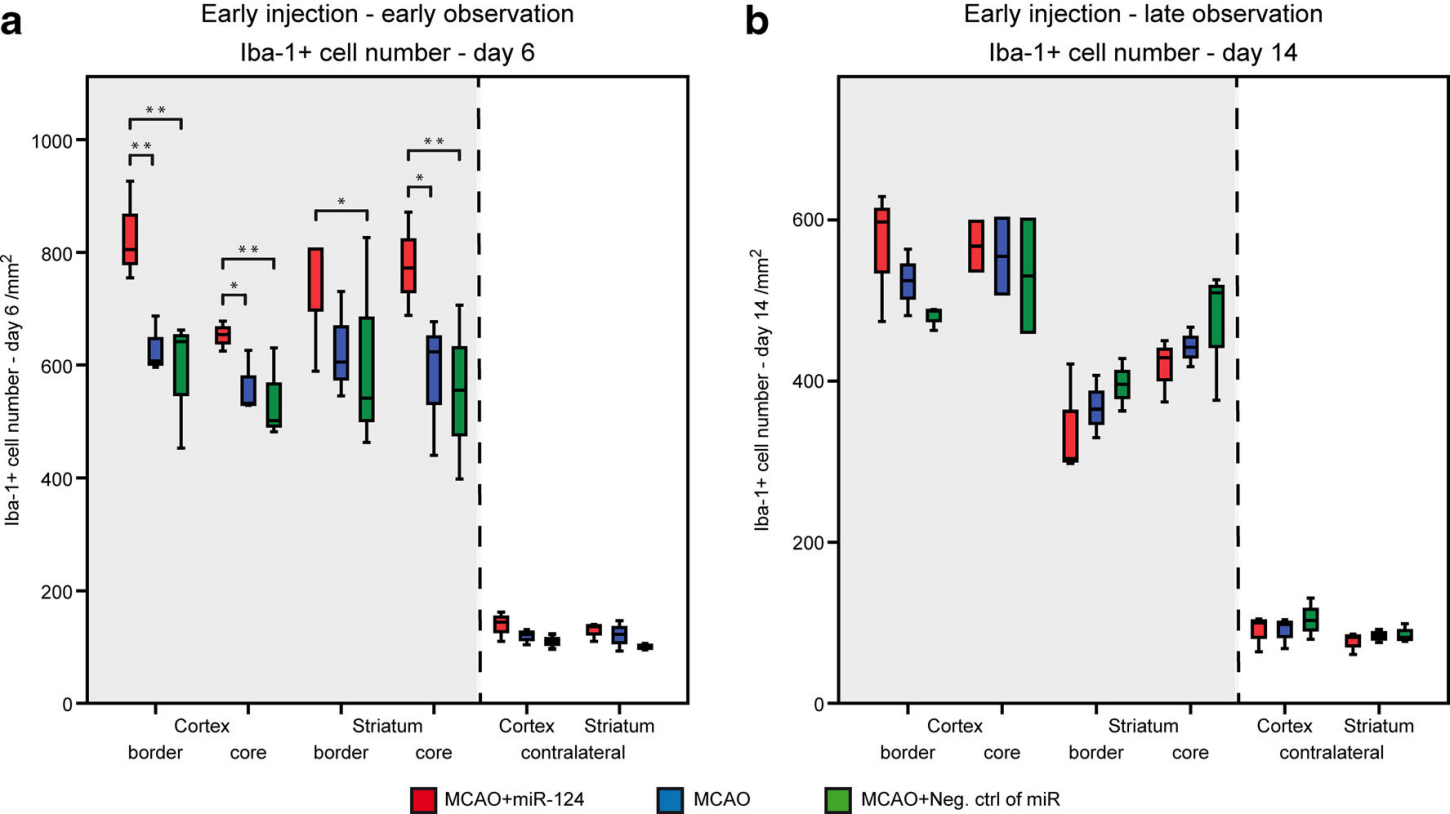
miR-124 effects microglial/macrophage kinetic after stroke. Densities of Iba-1 positive cells in the border and core zone of cortex and striatum area in miR-124 treated group comparing to the only MCAO and negative control group at day 6 (**a**) and day 14 after stroke (**b**). A significant increase in Iba-1 cell number in miR-124 treated mice was detected at day 6 after stroke. No significant difference in number of Iba-1 positive cells was found between the groups at day 14 after stroke. *n* = 5–6 mice in each group and for each time point. Median and minimum/maximum values are shown; *P < 0.05, **P < 0.01 at one-way ANOVA (with Bonferroni’s post hoc test)

We further evaluated the influence of liposomated miR-124 (injection at day 2 after MCAO) on the expression of Iba-1 at different time points after MCAO (Immunohistochemical analysis at day 6 Fig. 3a and day 14 Fig. 3b). Intracerebral injection of miR-124 at day 2 after stroke led to a significant increase in the number of Iba-1 cells. No significant difference in the number of Iba-1 positive cells was found between the MCAO-only and negative control group. At day 6 post MCAO, all ipsilateral interquartile ranges of Iba-1^+^ cell numbers were higher in the miR-124 treated group compared to both, the MCAO-only and the negative control group (Fig. 3a).

To observe the long term effects of the early injection of miR-124 on microglia/macrophage cells after stroke, we evaluated the Iba-1 cell number of the second experimental time point, where miR-124 or the random primer was injected 2 days after MCAO and animals were perfused at day 14 for immunohistochemical analysis. The density of Iba-1^+^ cells at day 14 markedly decreased in all 3 groups in all ipsilateral regions of interest, in comparison to the situation at day 6 post stroke (Fig. 3a, b). The Iba-1^+^ cell number in the cortical ROIs are at closely the same level in the three experimental groups (Fig. 3b).

### Modulation of Microglia/Macrophage Polarization by Early miR-124 Application

**Fig. 4:**
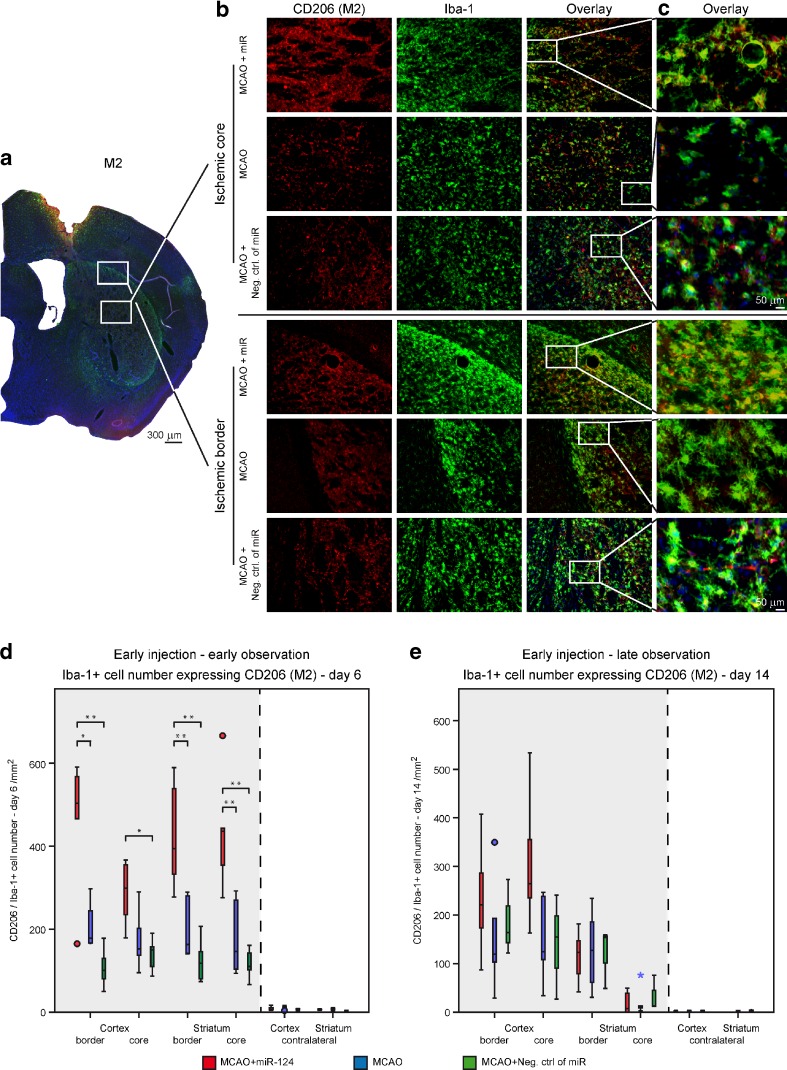
Effect of miR-124 on M2 phase of microglia/macrophages. Photomicrographs show examples of double-staining immunofluorescence of CD206 and Iba1 on brain sections of ischemic core and border zones acquired from three experimental groups at 6 days after stroke. Illustrative 4×, 20× close-up magnification visualize the changes in CD206 expression by Iba-1 positive cells (**a**-**c**). *Scale bar*: 50 μm. Time course for the CD206 expression by Iba-1+ cells in the ipsilateral and contralateral ROIs of cortex and striatum in miR-124 treated group comparing to the only MCAO and negative control group at day 6 (**d**) and day 14 (**e**). Quantification results confirm that intracranial injection of miR-124 at day 2 after MCAO results in a vigorous increase of Iba-1+ cells expressing CD206 monitored at day 6 post stroke (**d**). Representative graph shows no differences of CD206 expression by Iba-1+ cells at day 14 after stroke between groups (**e**). n = 5–6 mice in each group and for each time point. *P < 0.05, **P < 0.01 at one-way ANOVA (with Bonferroni’s post hoc test). *Scale bar* 50 μm

**Fig. 5:**
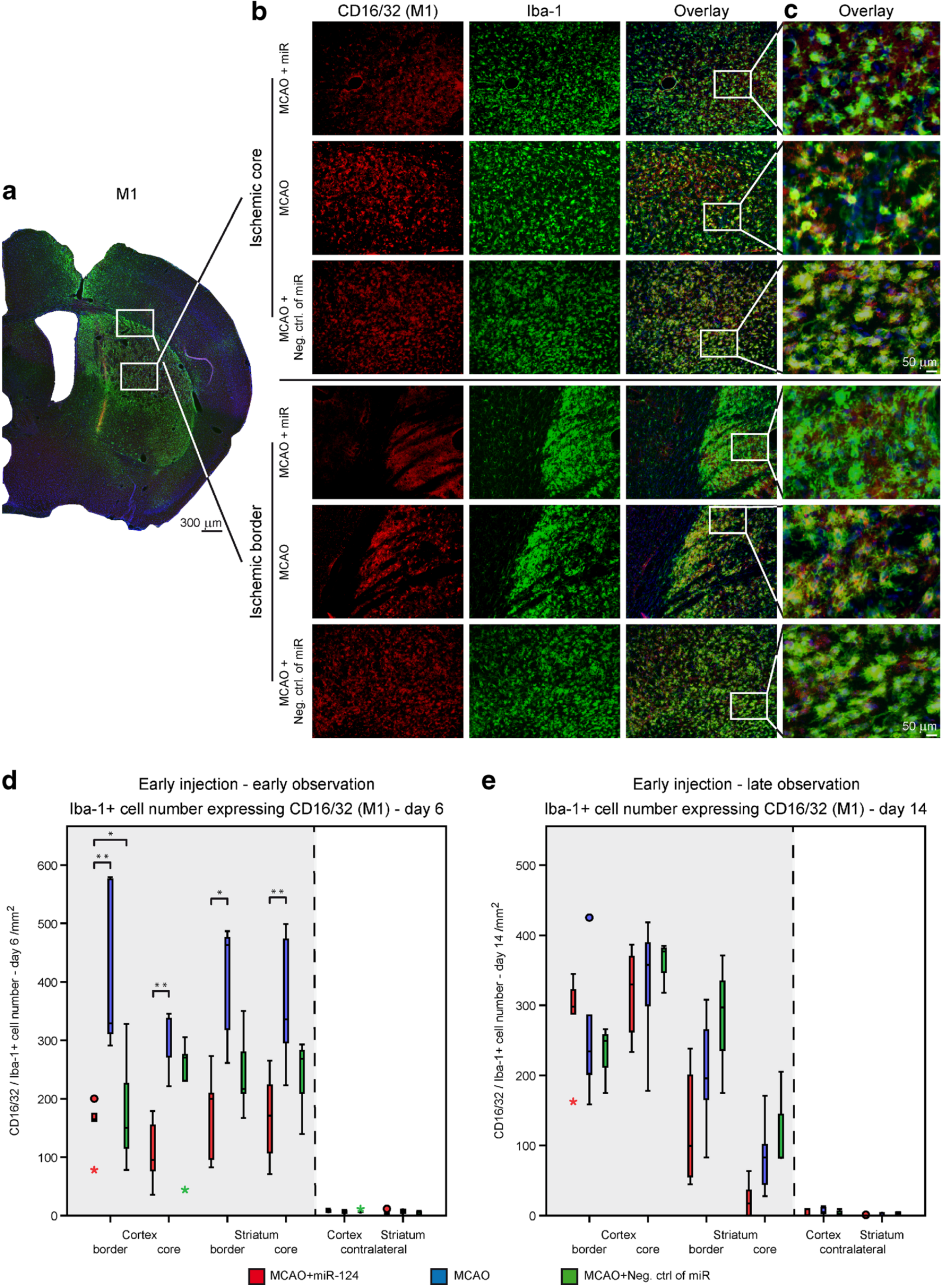
Effect of miR-124 on M1 phase of microglia/macrophages. Photomicrographs show examples of double-staining immunofluorescence of CD16/32 and Iba-1 on brain sections of ischemic core and border zones acquired from three experimental groups at 6 days after stroke. Illustrative 4×, 20× - close-up magnifications visualize the changes in CD16/32 expression by Iba-1 positive cells (**a**-**c**). *Scale bar*: 50 μm. Time course for the CD16/32 expression by Iba-1+ cells in the ipsilateral and contralateral ROIs of cortex and striatum in miR-124 treated group compared to the only MCAO and negative control group at day 6 (**d**) and day 14 (**e**). Quantification results confirm that intracranial injection of miR-124 at day 2 after MCAO results in a significant decrease of Iba-1+ cells expressing CD16/32 monitored at day 6 post stroke (**d**). Representative graph shows no differences at CD16/32 expression by Iba-1+ cells at day 14 after stroke between groups (**e**). *n* = 5–6 mice in each group and for each time point. *P < 0.05, **P < 0.01 at one-way ANOVA (with Bonferroni’s post hoc test). *Scale bar* 50 μm

Microglia/macrophages exhibit dynamic polarization over time, transforming from alternatively activated M2 phenotype to classical, activated M1 phenotype. We evaluated the characteristic polarization of microglia/macrophages after MCAO, using the representative M1-associated (CD16/32) or M2-associated (CD206) markers for double immunofluorescent staining together with Iba-1 in the ischemic territory (Fig. 4 for CD206; Fig. 5 for CD16/32). In both, the MCAO-only and negative control group, the expression of M1 marker CD16/32 in Iba-1^+^ cells was high at day 6 after stroke and remained elevated until day 14 after ischemia (Fig. 5d, e). In contrast, the immunofluorescence for the M2 marker CD206 was slightly decreasing over time from day 6 until day 14 in both groups (Fig. 4d, e).

Intracerebral administration of 100 ng miR-124 at day 2 after MCAO resulted in a strongly significant increase of Iba-1^+^ cells expressing CD206 at day 6 post stroke. Interestingly, at this time point, the M2 marker expression tends to be higher in the border areas than in the core of the ischemic lesion in the miR-124 treated group, emphasizing the anti-inflammatory role of miR-124 in the penumbra (Fig. 4d). However, in comparison of the three experimental groups, no significant long-term effects of miR-124 on M2 marker expression were noted, although the miR treated group showed larger interquartile range in the cortex areas of the ischemic hemisphere indicating a trend towards a higher amount of CD206 expression by Iba-1^+^ cells at day 14 after MCAO (Fig. 5e, Suppl. Fig. 2).

The immunofluorescence staining for M1 marker CD16/32 on Iba-1^+^ cells decreased significantly in the miR-124 treated group compared to both control groups at day 6 after MCAO. Almost all the relevant 50th percentile and the middle 50 % of the ipsilateral ROIs of this group were in lower level, indicating a noticeably lesser pro-inflammatory environment in this miR-124 treated group (Fig. 5d). With miR-124 treatment the M1 response increased strongly from 6 days to 14 days after MCAO, indicating the decreasing effect of miR-124 twelve days after injection (Fig. 5e).

Consequently, the early intracranial injection of miR-124 had a significant effect on the ratio of anti-inflammatory to pro-inflammatory phenotype in the first week after MCAO (Fig. 7).

### Effects of Delayed miR-124 Injection on M1/M2 Phenotype of Microglia/Macrophages

**Fig. 6:**
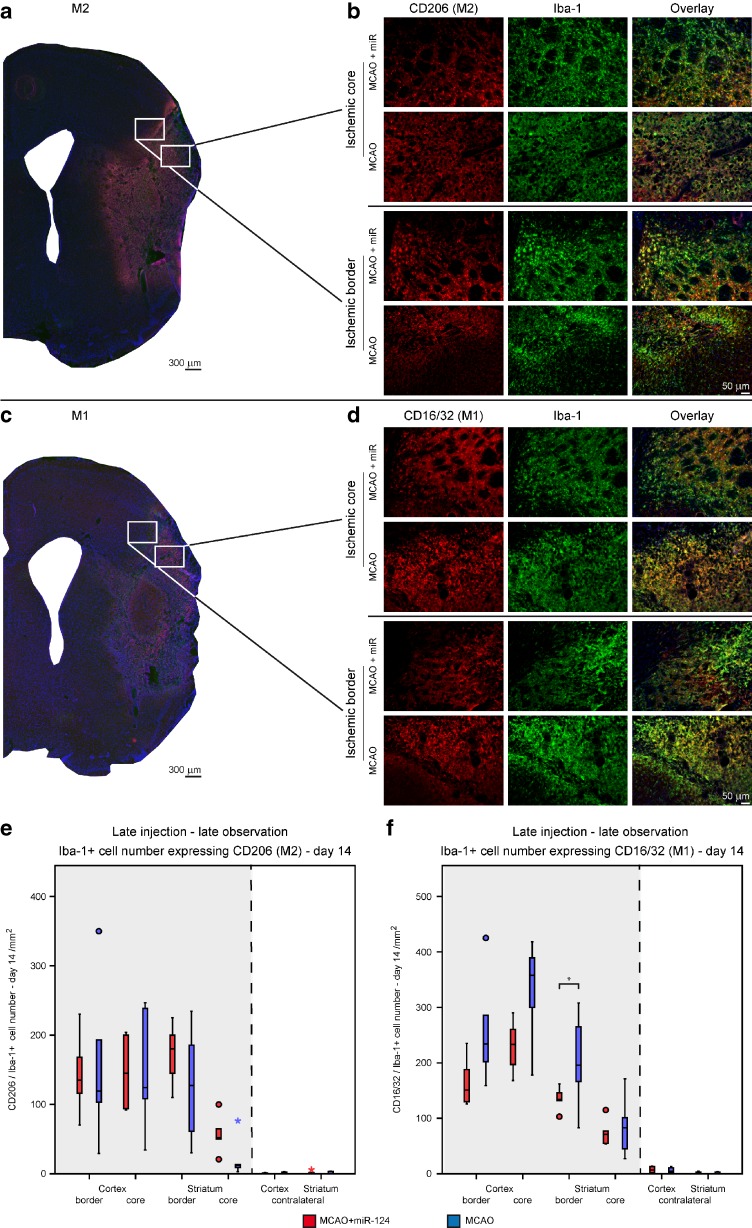
Effects of delayed miR-124 injection on M1/M2 phenotype of microglia/macrophages. Photomicrographs show examples of double-staining immunofluorescence of CD206 + Iba-1 (**a**-**b**) and CD16/32 + Iba-1 (**c**-**d**) on brain sections of ischemic core and border zones acquired from two experimental groups, where miR-124 was injected 10 days after MCAO and polarization of microglia/macrophages was analyzed 14 days after MCAO. Characteristic 4× and 20× magnifications display the changes in M2 and M1 markers expression by Iba-1 positive cells (**a**-**d**). *Scale bar*: 50 μm. Time course for the M2 (**e**) and M1 (**f**) markers expression by Iba-1+ cells in the ipsilateral and contralateral ROIs of cortex and striatum in miR-124 treated group comparing to the only MCAO group at day 14. Quantification results show no significant difference in the M2 expressing Iba-1+ cells number between the miR-124 treated and the only stroke groups (**e**). Representative graph confirms that intracranial injection of miR-124 at day 10 after MCAO results in pronounced decrease in M1 marker expression by Iba-1+ monitored at day 14 post stroke (f). *n* = 5 mice in each group.*P < 0.05, independent one-tailed Student’s t-test. Scale bar 50 μm

**Fig. 7:**
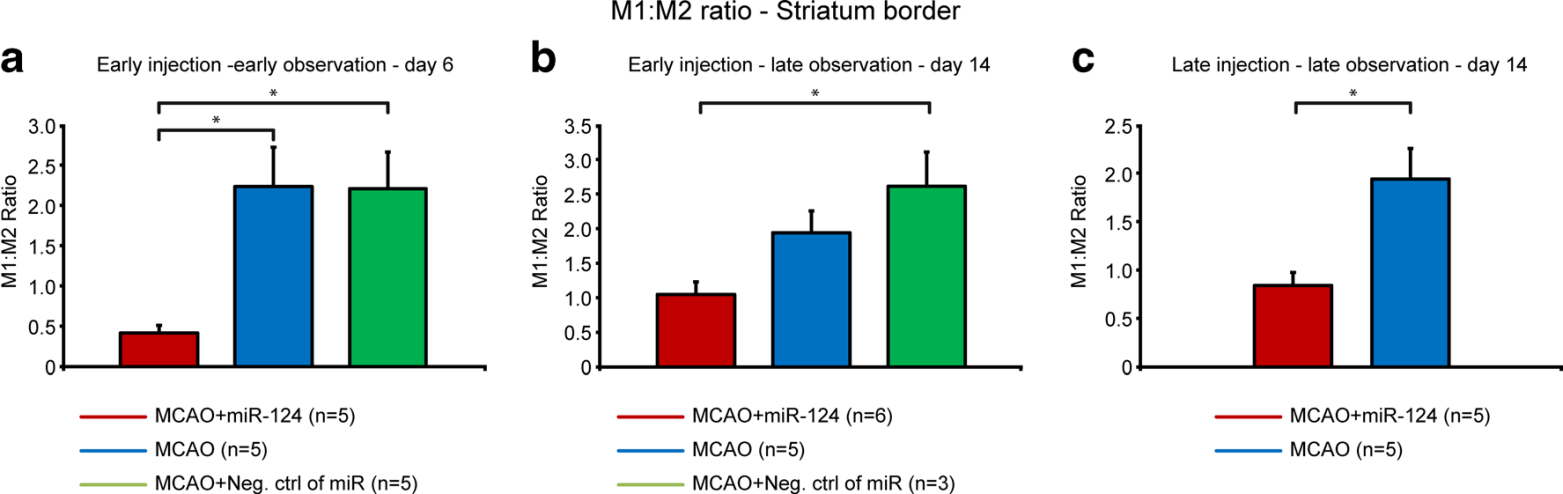
M1:M2 ratio in the ischemic border zone of the striatum. The ratio of cell number with M1 (CD16/32) and M2 (CD206) phenotype was determined for the ischemic border zone of the striatum for all three animal groups (mir-124 treated; negative control; MCAO-only). **a** and **b** show the data for miR-124 injection at day 2 after stroke and analysis at day 6 (**a**) and at day 14 (**b**). **c** depicts the situation for delayed miR-124 injection at day 10 and analysis 4 days later, at day 14. While the M1:M2 ratio of the miR-124 treated group is slowly increasing with longer period after injection and with later injection time after stroke, it remains always substantially lower than the control groups. Results are expressed as mean ± SEM. P < 0.05, as compared with control groups using Kruskal-Wallis H test for (**a**) and (**b**) and independent one-tailed Student’s t-test for (**c**)

We investigated whether miR-124 application can also shift between the M1 and M2 phenotype at a later time after stroke when the pro-inflammatory M1 phenotype has already become dominant. To survey its effects at this later stage of stroke evolution, miR-124 was injected 10 days after MCAO, and the polarization of microglia/macrophages was analyzed 14 days after MCAO. No significant difference was observed in the M2 expressing Iba-1^+^ cell number between the miR-124 treated and the stroke-only group (Fig. 6e). The median of both groups did not differ in the cortical regions, however the 50th percentile of the ipsilateral striatal regions of miR-124 treated group were higher, pointing towards slightly increased M2 marker expression by Iba-1^+^ cells adjacent to the injection channel (Fig. 6e). Interestingly, a pronouncedly lower level of M1 marker expression by Iba-1^+^ cells was clearly visible in the miR-124 treated group, particularly in the border and core regions of the striatal lesion (Fig. 6f). The coherent interquartile range and the 50th percentile of all ipsilateral ROIs in the miR-124 treated group are at a remarkably lower level confirming the reduced M1 to M2 ratio. Analyzing the M1:M2 ratio directly demonstrates a significantly lower ratio under miR-124 treatment in all experiments. While the M1:M2 ratio of the miR-124 treated group is increasing with time after application, indicating a slowly weakened treatment effect, it remains still significantly lower even when miR-124 is applied only after ten days (Fig. 7).

Taken together, we have demonstrated that the delayed miR-124 administration after stroke still can modify the microglia and macrophage polarization while early application of miR-124 administration after stroke is needed to achieve maximal increase of protective M2 in parallel with pro-inflammatory M1 decrease.

### Neurological Deficit Scores

**Fig. 8:**
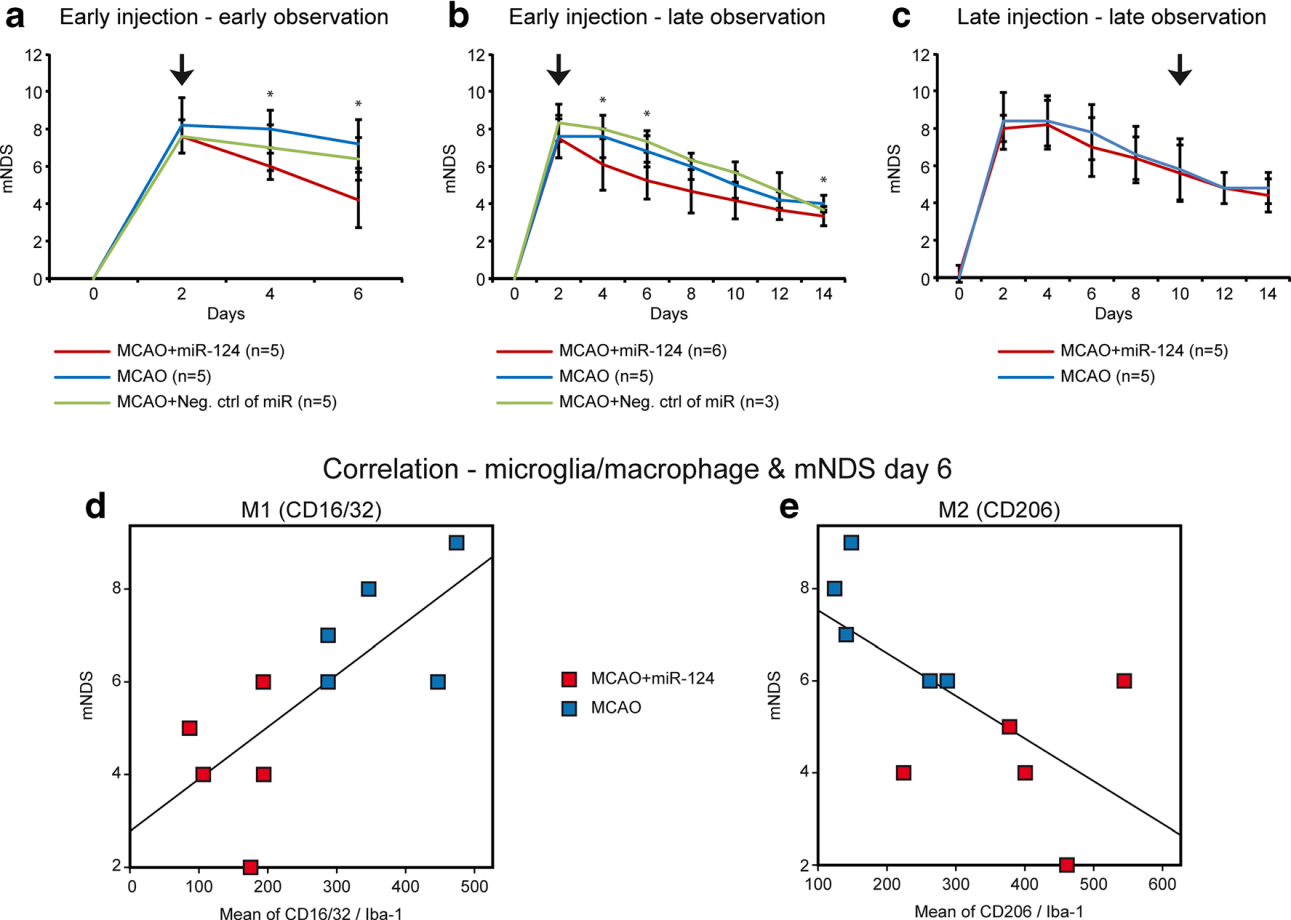
MiR-124 improves functional recovery after stroke. Behavioral functional test was evaluated by the modified neurological deficit scores (mNDS) before and after MCAO. Representative graphs of the experimental groups subjected to miR transplantation at 2 days after MCAO and perfused at day 6 (**a**) and day 14, respectively (**b**) show that miR-124 treatment at 2 days after MCAO significantly improved the functional recovery at day 4 and day 6 after stroke. Representative graphs of the experimental group subjected to miR transplantation at day 10 after MCAO with perfusion at day 14 (**c**) show no significant differences among the miR-124 treated group and only MCAO group. n = 5–6 mice in each group and for each time point. Values are mean ± SD. *P < 0.05, as compared with control groups using Kruskal-Wallis H test for (**a**) and (**b**), and Wilcoxon-Mann-Whitney test for (**c**). Changes of M1 expression within the ischemic hemisphere linearly correlates with neurological deficit score changes in the ischemic mice (Spearman r^2^ = 0.530, *p* = 0.017) (**d**). Changes of M2 expression within the ischemic hemisphere inversely correlates to neurological deficit score changes in the ischemic mice (Spearman r2 = 0.425, *p* = 0.041) (**e**)

We assessed whether intracerebral application of liposomated miR-124 improves functional recovery after stroke in mice, using the modified neurological deficit scores (mNDS). In all groups, 30 min MCAO increased the neurological deficit scores (up to 8 mNDS) compared to the days before stroke (0 mNDS) indicating impaired motor and sensory functions. Injection of miR-124 at two days after MCAO significantly reduced mNDS scores at 4 and 6 days (*P*˂0.05; 6 and 4 mNDS, respectively) compared with the stroke-only and negative control group (8 mNDS). Slight differences were observed between the stroke-only and negative control group, however the differences were not significant (Fig. 8a, b). The mice treated with liposomated miR-124 at day 10 showed no treatment effect on mNDS scores (Fig. 8c). Our findings clearly indicate that the neurological deficits caused by 30 min MCAO in mice can be improved by early miR-124 injection.

### Correlation of M1/M2 and NDS

Liposomated miR-124 treatment resulted in improved functional recovery, which was paralleled by the observation of decreased CD16/32 (M1) and increased CD206 (M2) expression of microglia/macrophages. We subsequently performed Spearman correlation analysis of M1 and M2 expression with the neurological deficit scores (Fig. 8d, e). Correlation analysis showed a positive linear relation between CD16/32 expression by Iba-1 positive cells and an increased neurological deficit score at day 6 after stroke with a linear equation NDS = 2.777 + 0.011 (CD16/32) (r^2^ = 0.530 , *p* = 0.017), (Fig. 8d). A negative linear correlation was found between CD206 expression by Iba-1 positive cells and an increased neurological deficit score at day 6 after stroke with an equation NDS = 8.455–0.009 (CD206) (r^2^ = 0.425, *p* = 0.041), (Fig. 8e). However, there was no correlation at day 14 post stroke.

## Discussion

The main finding of the present study is that local miR-124 injection after ischemic stroke improves the neurological score at day 4 and 6. The improved motor and sensory functions are directly correlated to the number of M2-like and inversely correlated to the number of M1-like microglia/macrophages. Taken together, our data present miR-124 as a promising molecule to modulate the microglia/macrophage activation state, and subsequently to improve the stroke recovery.

### Early Administration of miR-124, 2 days after Ischemic Stroke

In an effort to control the phenotypic shift of microglia/macrophages as a therapeutic strategy for brain disorders, we present a previously unreported role of the brain specific microRNA-124 in modulating the polarization of microglia and infiltrating macrophages in the sub-acute phase of ischemic stroke. We show here for the first time that miR-124 administration at 48 h after ischemia, before the pro-inflammatory peak of stroke (Hu et al. 2012), changes the M1/M2 balance toward a more anti-inflammatory phenotype and improves functional recovery at the first week after middle cerebral artery occlusion. The early miR-124 treatment at day 2 after ischemia gave rise to a strong increase of Iba-1^+^ cells expressing the M2 marker CD206 and a parallel pronounced decrease of the M1 marker CD16/32, highlighting the protective role of miR-124. In support of our experimental data, we observed a significant negative correlation between high M2 expression levels and neurological deficit scores in miR-124 administrated ischemic mice at day 6, paralleled by a significant positive correlation of the M1 level with neurological deficit scores.

Microglia/macrophages react dynamically to ischemic injury over time: in the acute phase the vast majority of recruited microglia and macrophages in CNS diseases are M2-dominant, providing protective functions, but by about 1 week after injury the M1 phenotype becomes predominant (Kigerl et al. 2009; Perego et al. 2011; Wang et al. 2013). The mRNA expression of M2 phenotype markers (CD206, Arg-1, Ym1/2, IL-10, and TGF-β) peaks between day 3 to day 5 post injury, while the M1 phenotype relevant genes (iNOS, CD16, CD32) are continuously increasing over time from day 3 on. They remain elevated still at day 14 after stroke (Hu et al. 2012; Pan et al. 2015). This is in agreement with the expression peak of IRF-4 at day 3, a key transcription factor of M2 polarization phenotype, whereas expression of IRF-8, a transcription factor of M1 polarization phenotype, increases within 72 h after ischemia (Guo et al. 2014; Xiang et al. 2014). The M2 phenotype promotes the CNS repair and regeneration, emphasizing that well timed counteraction of M2 on M1 should be a promising cell-based regenerative strategy in stroke.

We have shown that miR-124 enhances and prolongs the M2 phase while reducing the M1 process. Moreover, our results of improved functional outcome are in line with our earlier results, showing an upregulation of the M2-phenotype marker Arg-1 upon early administration of miR-124 after stroke (Hamzei Taj et al. 2016). In those studies, we could also find a negative correlation of Arg-1 expression with the neurological deficit score, paralleled by a positive correlation of Arg-1 cell expression with neuronal survival (Hamzei Taj et al. 2016). Further support for our finding of miR-124 induced improved functional outcome comes from Ponomarev (Ponomarev et al. 2011), who reported that bone marrow-derived macrophages, transfected with miR-124, downregulate the expression of the M1 markers TNF-α and inducible nitric oxide synthase (iNOS), and simultaneously upregulate the expression of the M2 markers TGF-β1, Arg-1 and FIZZ1 (Found in inflammatory zone 1) by inhibition of the C/EBP-α transcription factor responsible for downregulation of PU.1. A similar M1/M2 ratio modulation of microglia and macrophages in the spinal cord was achieved via intrathecal injection of liposomated miR-124 in an IL-1β induced hyperalgesia model, leading to reduced inflammatory reactions and neuropathic pain (Willemen et al. 2012). When transfecting primary microglia isolated from neonatal rats with miR-124/chitosan complex particles, expression of TNF-α and inducible NO synthetase (iNOS), two major substances of M1 phenotype, was reduced. Microinjection of miR-124/chitosan particles was also shown to reduce the inflammation in a rat model of spinal cord injury (Louw et al. 2015). On the other hand, knockdown of miR-124 via a miR-124 antisense oligonucleotide inhibitor was reported to activate the microglia and macrophages, presenting upregulated MHC class II and CD45 in addition to inhibiting the development of ramified morphology of the cells (Ponomarev et al. 2011).

It is worth noting that the induced modulation of immune response after stroke was mostly due to miR-124 injection in the present study and not because of intracranial injection procedure. Interestingly, however, the early injection of negative control of miR at day 2 after ischemia only gave rise to a smaller M1 marker expression by Iba-1^+^ cells in comparison to the stroke only group. In support of our observation, Le Blon and colleagues observed fewer MHCII^+^ expressions by Iba-1^+^ cells, as M1 associated marker, in sham implanted group in a mouse model of mesenchymal stem cell implantation (Le Blon et al. 2014).

The M1/M2 modulating effect of the liposomated miR-124 was decreasing from day 6 to day 14 in our experiment. Although the number of CD206^+^/Iba-1^+^ cells and CD16/32^+^/Iba-1^+^ cells of the miR-124 treated group are no longer statistically significantly different from the corresponding cell number of the control groups, a clear difference is still noted resulting in the significantly lower M1:M2 ratio of the treated group (cf Fig. 7). We suggest that the time dependent decrease of the miR-124 immunomodulatory effect is explained by the short half-life of microRNAs of a few days only (Krol et al. 2010; Gantier et al. 2011). With a different route of miR-124 delivery the long term effect may be extended or even enhanced. Thus, Doeppner et al. presented sustained neuroprotection and ameliorated motor coordination and memory acquisition up to 56 days upon stroke onset by a viral vector-mediated miR-124 delivery (Doeppner et al. 2013). In correspondence to that, our findings of a decreasing immunomodulatory effect after a single injection of liposomated miR-124 together with the still persistent effect upon delayed injection indicates that future protocols with repetitive injections of liposomated miR-124 may provide a sustained low M1:M2 ratio.

### Delayed Administration of miR-124, 10 days after Ischemic Stroke

We found that intracerebral injection of liposomated miR-124 at the chronic stage after stroke onset, administered at day 10, still has substantial influence on the microglia/macrophages. However, while at early miR-124 application M2 was enhanced and M1 in parallel decreased, late phase application only decreased the pro-inflammatory M1 phenotype but left the M2 marker expression unaffected. This is in full agreement with our own earlier observation, where we showed that Arg-1, an M2 marker like CD206 used in the present study, was upregulated upon early miR-124 administration, but that this upregulation was lost when miR-124 was applied after ten days (Hamzei Taj et al. 2016). The miR-124 administration at this late stage when the pro-inflammatory phase has become dominant (Hu et al. 2012) could not improve the neurological deficit. Modulating the neuroinflammation in neurodegenerative disorders as a therapeutic strategy is only now emerging, and to our knowledge this is the first study to analyze the effect of polarization modulation of microglia/macrophage cells by liposomated miR-124 at different time points after stroke.

The M1 polarization process gradually increases from day 3 onward after stroke onset (Hu et al. 2012). We therefore addressed the question whether the delayed administration of miR-124 still has an effect on modifying the M1/M2 polarization. The strength of neuroinflammation modulation (i.e. M1:M2 ratio) in response to late miR-124 administration was lower compared to application at the early stage, which caused a pronounced increase in microglia/macrophages population with M2 phenotype and a noticeable decrease in the population with M1 phenotype. At this late time point, we observed a considerable decrease in M1 surface marker expression by Iba-1^+^ cells particularly in the border and core regions of the striatal lesion by miR-124 administration. Furthermore, the higher level of 50th percentile of the M2 surface marker expression of the ipsilateral striatal regions in the miR-124 treated group shows a small tendency of increased M2 surface marker expression by Iba-1^+^ cells at this time point. Consequently, even late miR-124 administration still has a clear, although somewhat weaker, modulatory effect on the M1-M2 balance by reducing the M1:M2 ratio, thus producing a clear, persistent shift towards the anti-inflammatory, neuroprotective microglia/macrophage phenotype. Intrathecal injection of liposomated miR-124 administration during the first 15 h of carrageenan-induced inflammatory hyperalgesia, when the pro-inflammatory process is prominent in this disease model, could effectively reduce the M1 surface marker and attenuated the resistant thermal hyperalgesia, although the efficacy of miR-124 on M1/M2 polarization at later time points of persistent hyperalgesia was not found (Willemen et al. 2012). Treatment with minocycline, a selective inhibitor of M1 microglia (Kobayashi et al. 2013), at day 4 after stroke, improved neurogenesis and functional recovery (Liu et al. 2007). These results suggest that targeting the correct phenotype of immune cells at the right time of neurodegenerative disorders is important for a therapeutic effect.

Our data demonstrate that miR-124 works in the early stage of stroke by modulating the microglia/macrophage activation towards M2 phenotype, leading to improved functional recovery. Delayed miR-124 administration after stroke still modified the microglia and macrophages M1/M2 polarization ratio, however with reduced intensity. This indicates that a maximal effect is achieved for miR-124 administration before the dominance of the pro-inflammatory process of stroke, i.e. within the first few days when the anti-inflammatory phase of the M2 phenotype is dominant. Moreover, therapeutic effect of the long term observed polarization shift by miR-124 was limited to early application. It clearly emphasizes the necessity of careful adjustment of the polarization balance of microglia/macrophages in therapeutic approaches targeting neuroinflammation. Although much work is still needed before clinical translation, our findings provide important steps for the understanding of microRNA based therapy and the underlying mechanisms to develop microRNA injection into a new approach for functional improvement after ischemic stroke.

## Conclusions

In conclusion, our study demonstrates that a single injection of miR-124 into the mouse brain in the early phase after stroke is sufficient to shift the M1/M2 balance of immune cells towards the anti-inflammatory phenotype which correlates with behavioral improvement. Further studies are needed to clarify the mechanism and identify the targeted immune cells. Nevertheless, our findings highlight the important role of immune cells after stroke and the therapeutic relevance of their polarization balance. As there is no treatment for sub-acute stroke available, miR-124 evolves as a promising novel therapeutic approach.

CCA, common carotid artery; C/EBP-α, CCAAT enhancer binding protein α; CNS, central nervous system; ECA, external carotid artery; FIZZ, found in inflammatory zone; Iba-1, ionized calcium-binding adapter molecule 1; ICA, internal carotid artery; IFN, interferon; IL, interleukin; iNOS, inducible NO synthetase; IQR, interquartile range; KPBS, potassium phosphate buffered saline; MCAO, middle cerebral artery occlusion; MHC, major histocompatibility complex; MMR, mouse anti-mannose receptor; mNDS, modified neurological deficit score; mRNA, messenger ribonucleic acid; MRI, magnetic resonance imaging; MSME, multi slice multi echo; PBS, phosphate buffered saline; PFA, paraformaldehyde; RF, radio frequency; RNA, ribonucleic acid; siRNA, small interfering ribonucleic acid; TGF-β, tumor growth factor β; TNF-α, tumor necrosis factor α.

## Electronic supplementary material

**Supplementary 1 – Quantitative analysis of the T2 based ischemic lesion volume.** Quantitative analysis ischemic lesion volume determined on the elevated T2 relaxation time of the T2 MRI shows no significant didfference between groups for all three experimental protocols. (AI 294 kb)

**Supplementary 2 – M1/M2-Day 14**. Photomicrographs show examples of double-staining immunofluorescence of CD206 + Iba-1 (a-b) and CD16/32 + Iba-1 (c-d) on brain sections of ischemic core and border zones acquired from three experimental groups, where miR-124 was injected 2 days after MCAO and polarization of microglia/macrophages was analyzed 14 days after MCAO. Characteristic 4× and 20× magnification display no significant changes in M2 and M1 markers expression by Iba-1 positive cells (a-d). Scale bar: 50 μm. *n* = 5–6 mice in each group. (GIF 505 kb)

High resolution image (TIFF 30013 kb)
