# Supplementary figures and images for: Dynamic Modulation of Microglia/Macrophage Polarization by miR-124 after Focal Cerebral Ischemia
Source: J Neuroimmune Pharmacol. 2016 Aug 18;11(4):733–48. doi: 10.1007/s11481-016-9700-y (PMC5097787; doi:10.1007/s11481-016-9700-y)

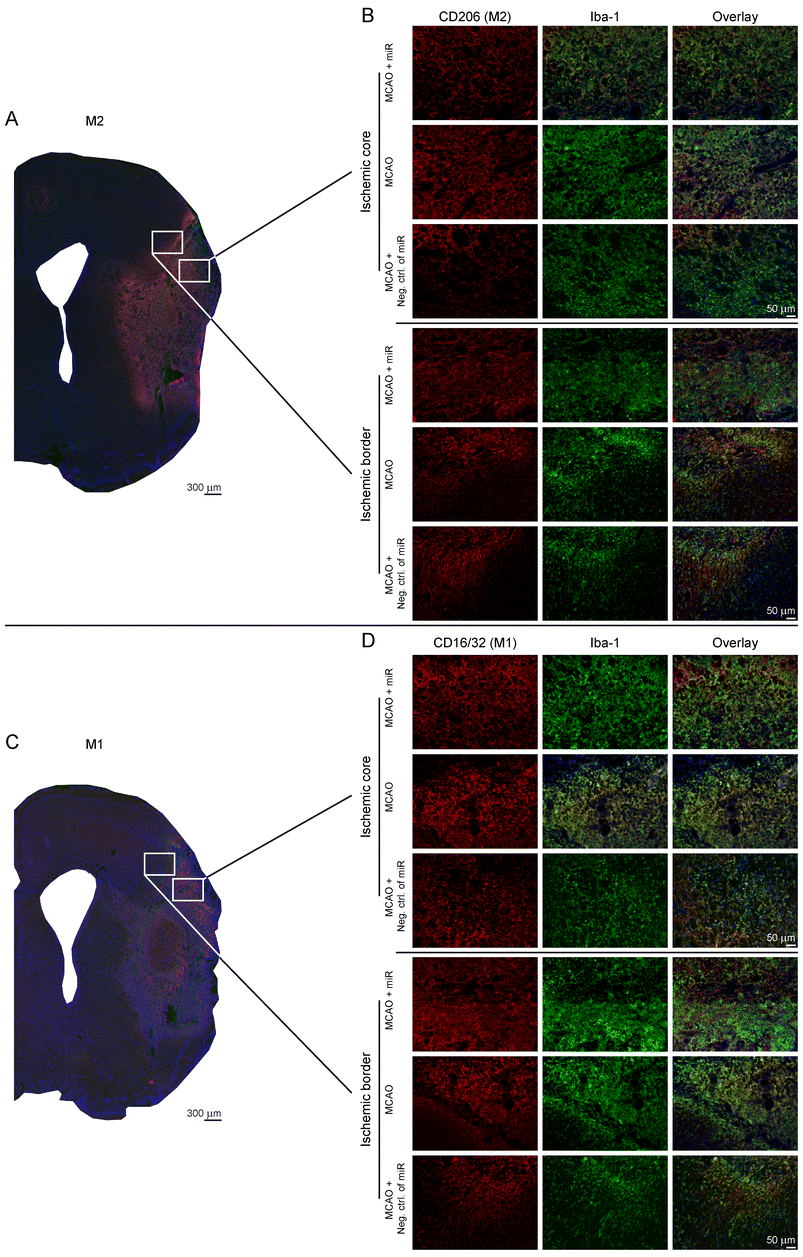

Supplement: Supplementary file 2 — Supplementary 2 – M1/M2-Day 14. Photomicrographs show examples of double-staining immunofluorescence of CD206 + Iba-1 (a-b) and CD16/32 + Iba-1 (c-d) on brain sections of ischemic core and border zones acquired from three experimental groups, where miR-124 was injected 2 days after MCAO and polarization of microglia/macrophages was analyzed 14 days after MCAO. Characteristic 4× and 20× magnification display no significant changes in M2 and M1 markers expression by Iba-1 positive cells (a-d). Scale bar: 50 μm. n = 5–6 mice in each group. (GIF 505 kb) [file 11481_2016_9700_Fig9_ESM.gif]

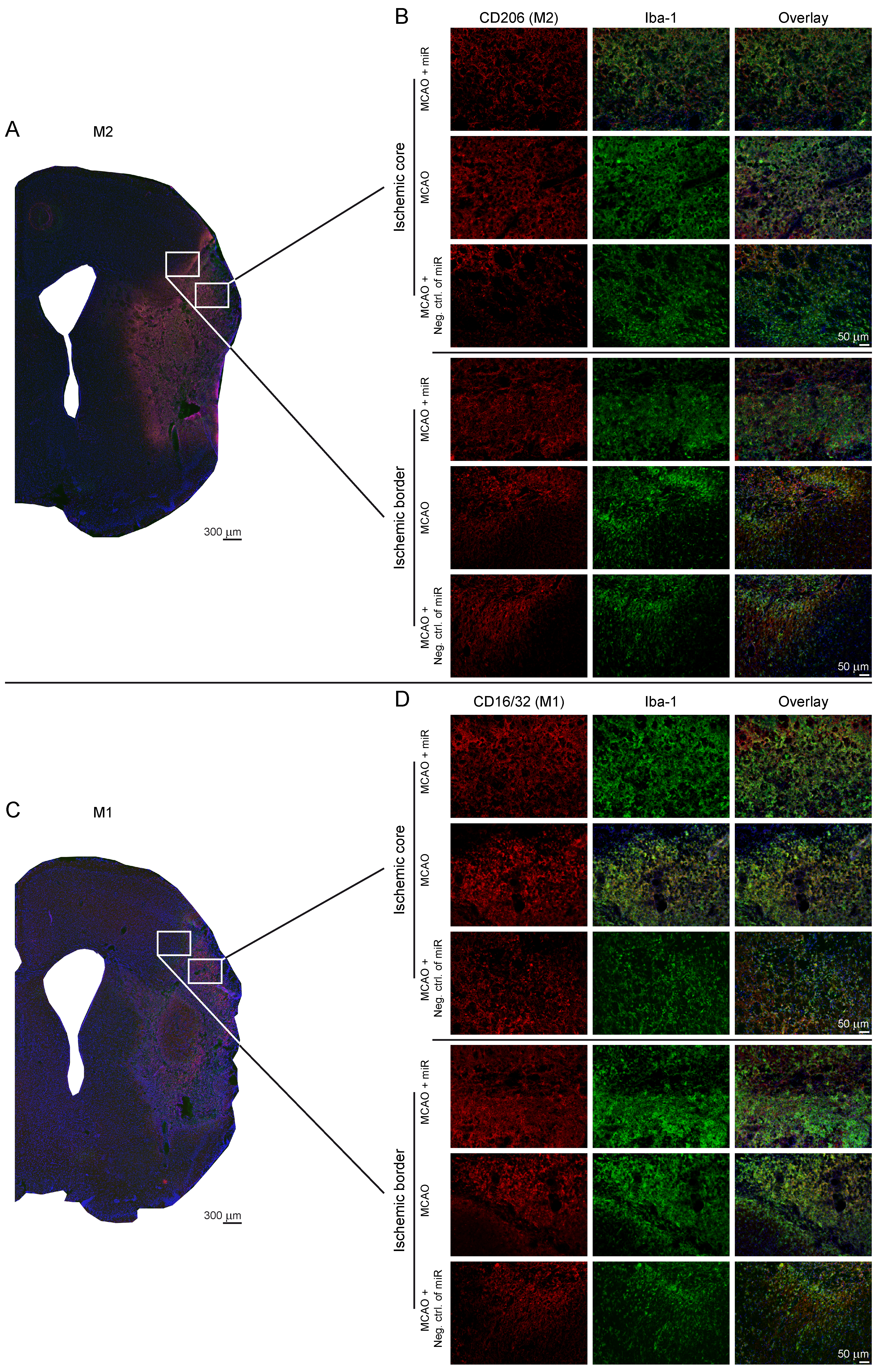

Supplement: Supplementary file 3 — High resolution image (TIFF 30013 kb) [file 11481_2016_9700_MOESM2_ESM.tif]
